# Dietary n-3 Polyunsaturated Fatty Acids in Late Pregnancy and Postpartum Depressive Symptom among Japanese Women

**DOI:** 10.3389/fpsyt.2017.00241

**Published:** 2017-11-23

**Authors:** Minatsu Kobayashi, Kohei Ogawa, Naho Morisaki, Yukako Tani, Reiko Horikawa, Takeo Fujiwara

**Affiliations:** ^1^Department of Food Science, Faculty of Home Economics, Otsuma Women’s University, Tokyo, Japan; ^2^Center for Maternal-Fetal, Neonatal and Reproductive Medicine, National Center for Child Health and Development, Tokyo, Japan; ^3^Department of Social Medicine, National Research Institute for Child Health and Development, National Center for Child Health and Development, Tokyo, Japan; ^4^Department of Global Health Promotion, Tokyo Medical and Dental University (TMDU), Tokyo, Japan; ^5^Japan Society for the Promotion of Science, Tokyo, Japan; ^6^Division of Endocrinology and Metabolism, National Center for Child Health and Development, Tokyo, Japan

**Keywords:** fish intake, dietary n-3 polyunsaturated fatty acids, eicosapentaenoic acid, docosahexaenoic acid, postpartum depression, Japanese pregnant women

## Abstract

**Background:**

The use of n-3 polyunsaturated fatty acids (n-3PUFA) in preventive or therapeutic modalities for postpartum depression, especially long-chain types such as eicosapentaenoic acid (EPA) and a docosahexaenoic acid (DHA), is of considerable interest. High n-3PUFA consumption has been reported among pregnant Japanese women. Therefore, analysis of this group could provide important insights into the relationship between postpartum depression and dietary n-3PUFA consumption. To further examine the relationship between the risk of postpartum depression and n-3PUFA consumption, we conducted a prospective hospital-based birth cohort study in Japan.

**Design and methods:**

Our prospective birth cohort study was performed at the National Center for Child Health and Development (NCCHD) in suburban Tokyo, Japan. Dietary n-3PUFA intake during late pregnancy was assessed by a semi-quantitative food questionnaire and participants were categorized by quintile distributions of n-3PUFA intake. A Japanese translation of the Edinburgh Postnatal Depression Scale was used to screen women for postpartum depression at 1 month after delivery (967 women) and at 6 months after delivery (710 women). We performed logistic regression analysis to examine the relationship between the risk of postpartum depression and n-3PUFA consumption after adjusting for confounding factors.

**Results:**

Significant associations between EPA, DHA, and n-3PUFA intakes in late pregnancy and postpartum depression at both 1 and 6 months after delivery were not observed.

**Conclusion:**

This prospective study indicated that EPA, DHA, and n-3PUFA intake during late pregnancy was not associated with the risk of postpartum depression.

## Introduction

Postpartum depression is one of the most common psychopathologies with a reported prevalence between 10 and 15% (1). The condition is associated with long-term risks for women’s mental health and significant negative effects on the cognitive, social, and physical development of their children (2). Considerable interest has been shown in the potentiality of n-3 polyunsaturated fatty acids (n-3PUFA), especially long-chain ones such as an eicosapentaenoic acid (EPA) and a docosahexaenoic acid (DHA), to prevent depression (3–11). Moreover, these fatty acids could provide preventive or therapeutic modalities for postpartum depression by limiting the production of pro-inflammatory eicosanoids and cytokines and may be desirable because they provide an alternative to major tranquilizers, which may be toxic to the fetus (12). n-3PUFAs also help regulate the production, function, and metabolism of serotonergic neurotransmitters (13–15). However, reports of epidemiological studies on the relationship between postpartum depression and n-3PUFA consumption during pregnancy estimated from dietary surveys (16, 17) or n-3PUFA levels of biological information (18, 19) showed no associations. Furthermore, although several randomized control studies examined the relationship between postpartum depression and n-3PUFA supplementation during pregnancy, no evidence was reported to support the routine use of n-3 supplementation during pregnancy (20–23).

It is well known that pregnant Japanese women eat a wide range of n-3PUFA sources (24, 25). Therefore, pregnant Japanese women can be regarded as suitable participants for analyzing the relationship between postpartum depression and n-3PUFA consumption during pregnancy. Although one study on this relationship has already been conducted in Japan, the dietary survey used in the study to collect information on fish and PUFA intakes did not specify gestational week and the timeframe for the Edinburgh Postnatal Depression Scale (EPDS) was quite wide (16). To further examine the relationship between the risk of postpartum depression and n-3PUFA consumption, we conducted a prospective cohort study in Japan.

## Materials and Methods

### Study Population

Our prospective birth cohort study was performed at the National Center for Child Health and Development (NCCHD) in suburban Tokyo, Japan. Enrollment of pregnant women in the cohort occurred over a period of 3 years and 6 months, from May 13, 2010 until November 28, 2013. Participants were recruited during their first antenatal visits, which usually take place in gestational weeks’ 6–14. Medical records and anthropometric measurements for pregnant women and children were retrieved from hospital charts for all deliveries. A questionnaire including demographic data such as socioeconomic and lifestyle factors and a semi-quantitative food frequency questionnaire (sFFQ) was administered in gestational weeks’ 26–40. Psychiatric data were obtained at 1 and 6 months after delivery using a Japanese translation of the EPDS.

**Figure 1:**
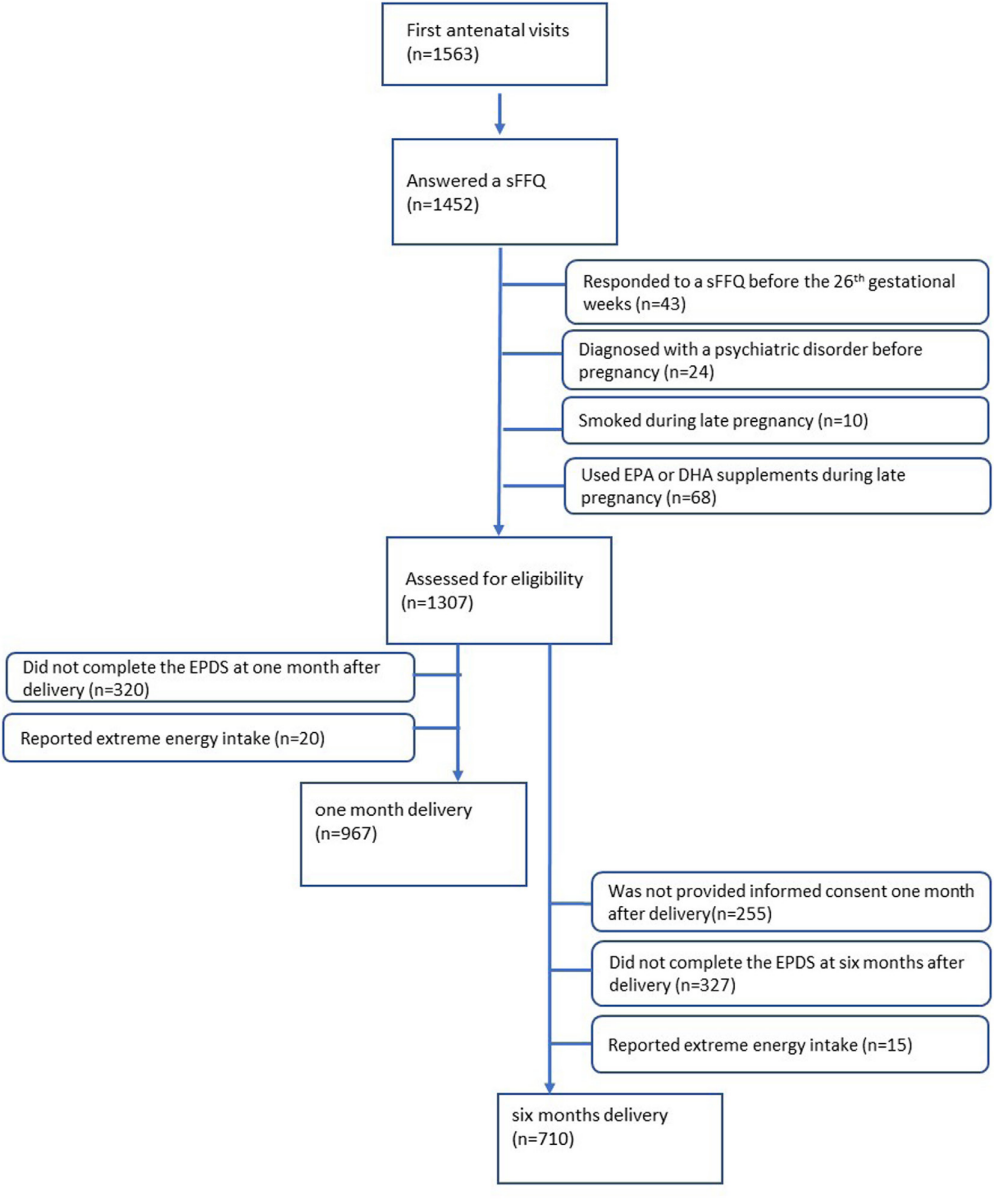
Subject flow diagram.

Of the 1,563 women who provided informed consent at their first antenatal visits, 1,452 women answered the sFFQ (response rate: 92.9%) and indicated consumption of fatty acids during mid- to late pregnancy. The exclusion criteria are as follows: women who responded to a sFFQ before the 26th gestational week (*n* = 43), women who were diagnosed with a psychiatric disorder before pregnancy (*n* = 24), women who smoked during late pregnancy (*n* = 10), and women who used EPA or DHA supplements during late pregnancy (*n* = 68). Of the 1,307 women eligible for assessment, 320 women did not complete the EPDS at 1 month after delivery, and 20 women reported extreme energy intake (top and bottom 1.0%). Therefore, 967 women were included in the analysis at 1 month after delivery. At 6 months after delivery, of the 1,307 women eligible for assessment, 255 women did not provide informed consent 1 month after delivery, 327 who did not complete a questionnaire at 6 months after delivery, and 15 reported extreme energy intake (top and bottom 1.0%) were excluded. Finally, 710 women were included in the analysis at 6 months after delivery (Figure 1). Although the same 642 women were included in the analysis both at 1 and 6 months after delivery, we used the sample of 710 women to increase statistical power. All procedures performed in this study were conducted in accordance with the ethical research standards on the institutional and national research ethics committee and with the 1964 Helsinki declaration of 1964. The study protocol was approved by the Ethics Committee at the National Center for Child Health and Development on August 2, 2010 (project number 417).

### Assessment of Exposure

The sFFQ included 165 food items and nine frequency categories and asked participants about the habitual consumption of listed foods within the past 2 months (26). We calculated fish consumption based on the amount of 20 fish items consumed per day and then computed the total energy intake, and calculated each fatty acid intake per day using a food composition table developed for the sFFQ based on the Standardized Tables of Food Composition in Japan (2010 edition) (27). Fish and fatty acid intakes were adjusted by total energy intake with a residual model.

Validation of the sFFQ in computing total energy and n-3PUFA was assessed using the 3-day dietary records of 188 participants and their serum phospholipid levels. Spearman rank correlation coefficients between the sFFQ and the dietary records were 0.38 for EPA and 0.40 for DHA, and those between the sFFQ and serum phospholipid levels were 0.41 for EPA and 0.35 for DHA (26, 28).

### Measurement of Postpartum Depression

A version of the EPDS translated into Japanese was used to screen women for postnatal depression, at 1 and 6 months after delivery. The EPDS is a 10-item self-report screening tool for postnatal depression. Each item is scored on a 4-point scale ranging from 0 to 3. As total scores can range from 0 to 30 and the cutoff value of ≥9 has been shown to indicate good sensitivity and specificity for postpartum Japanese women (29), we defined postpartum depression as an EPDS score of 9 or over.

### Statistical Analysis

Initially, data were expressed as mean ± SD for continuous variables and a percentage for categorical variables. Differences in continuous variables among postnatal depression cases and controls were tested using the *t*-test whenever the variables had a normal distribution, while the Mann–Whitney was used for those with a skewed distribution. Differences in categorical variables among postnatal depression cases and controls were tested using the chi-square test whenever the expected values in any of the cells of a contingency table were above five, while Fisher’s exact test was used for those cells below five.

Eicosapentaenoic acid, DHA, and total n-3PUFA consumption were assessed by categories of quintile distribution. We performed logistic regression analysis to examine the relationship between risk of postpartum depression and n-3PUFA consumption using EPDS scores with a cutoff of nine points as the dependent variable, and EPA, DHA, total n-3PUFA, total n-6PUFA consumption and the ratio of n-3PUFA to n-6PUFA at 1 and 6 months after delivery as the independent variable. Finally, adjustments were made for possible confounding factors, including age (<30, 30–35; 35–40, and ≥40 years), pre-pregnancy BMI (<18.5, 18.5–25, and ≥25), marital status (yes, no), normal spontaneous delivery (yes, no), multiple fetuses (yes, no), parity (0 or ≥1), gestation period (before 37 weeks, after 37 weeks), baby’s sex (male or female), maternal educational background (high school or less, graduated college, and graduated university), annual income (<4 million, 4–8 million, and ≥8 million yen), and psychological distress during mid-pregnancy diagnosed with a Kessler-6 score (cut-off value of ≥9, <9) (30). These variables are either known or suspected from previous studies as risk factors for postpartum depression (31, 32). All analyses were conducted using the statistical software package Stata 14 (STATA Corp., College Station, TX, USA), and *p*-value <0.05 was considered as statistically significant when performing hypothesis tests.

## Results

At 1 month after delivery, 19.8% (191/967) of participants reported significant depression (EPDS ≥9). The respective prevalence at 6 months after delivery was 12.8% (91/710). Lifestyle and background characteristics of cases and controls at 1 month after delivery and 6 months after delivery are shown in Tables 1 and 2, respectively. Although women with postpartum depression at 6 months had a higher energy intake (*p* = 0.011), differences were not observed for fish intake and PUFA consumption both at 1 and 6 months. Women with postpartum depression were more likely to experience psychological distress (Kessler-6 score ≥9) both at 1 month (*p* < 0.001) and 6 months (*p* < 0.001) postpartum. Women with a singleton pregnancy and multiparous women had a lower risk of postpartum depression only at 1 month after delivery. Women with a higher household income had a lower risk of postpartum depression both at 1 and 6 months.

**Table 1:** Characteristics of participants with and without depression [Edinburgh Postnatal Depression Scale (EPDS) score ≥9] at 1 month after childbirth.

		EPDS score ≥9 (*n* = 191)	EPDS score <9 (*n* = 776)	*p*-Value
			
		(Mean ± SD)	(Mean ± SD)	
Maternal age	year	35.7 ± 4.5	36.1 ± 4.1	0.319
Pre-pregnancy BMI		20.5 ± 2.9	20.6 ± 2.6	0.609
Energy intake	kcal	1,813.4 ± 634.3	1,768.7 ± 610.1	0.368
Fish intake	g	35.1 ± 30.1	34.5 ± 21.2	0.306
Eicosapentaenoic acid intake	mg	152.9 ± 172.2	141.8 ± 98.8	0.394
Docosahexaenoic acid intake	mg	238.4 ± 232.8	228.8 ± 154.8	0.317
n-3 PUFA intake	g	2.1 ± 0.8	2.1 ± 0.6	0.927
n-6 PUFA intake	g	11.6 ± 2.7	11.6 ± 2.8	0.940
n-3 PUFA/n-6 PUFA		18.3 ± 4.7 (%)	18.1 ± 3.7 (%)	0.739
**Marital status**																				
Married		98.4	99.0	0.597
**Psychological distress at mid-pregnancy^a^**																				
Yes		77.0	51.3	<0.001
**Alcohol intake**				
>once/month		2.6	4.1	0.617
**Annual income (yen)**																				
Less than 4 million		12.0	6.6	0.001
4–8 million		40.3	31.2	
Over 8 million		39.8	53.7	
Missing		7.9	8.5	
**Maternal education**															
Training school, high school, or less		23.0	16.2	0.096
Graduated college		18.9	17.1	
Graduated university		55.5	62.6	
Missing		2.6	4.0	
**Experience of giving birth**																				
Yes		18.9	43.0	<0.001
**Normal delivery**																				
Yes		51.3	53.0	0.807
**Multiple fetuses**																				
Yes		4.7 21	1.7	0.025
**Gestation period**			
Before 37 weeks		3.1	4.8	0.434

*^a^Diagnosed with Kessler-6 score (cutoff value of ≥9)*.

**Table 2:** Characteristics of participants with and without depression [Edinburgh Postnatal Depression Scale (EPDS) score ≥9] at 6 months after childbirth.

		EPDS score ≥9 (*n* = 91)	EPDS score <9 (*n* = 619)	*p*-Value
			
		(Mean ± SD)	(Mean ± SD)	
Maternal age	year	36.1 ± 4.1	36.4 ± 4.1	0.601
Pre-pregnancy BMI		21.0 ± 3.0	20.4 ± 2.6	0.102
Energy intake	kcal	1,935.4 ± 660.9	1,766.8 ± 573.6	0.011
Fish intake	G	34.8 ± 23.2	34.1 ± 21.1	0.779
Eicosapentaenoic acid intake	mg	145.3 ± 107.0	140.6 ± 98.0	0.672
Docosahexaenoic acid intake	mg	237.7 ± 168.7	227.2 ± 153.8	0.549
n-3 PUFA intake	g	2.1 ± 0.6	2.1 ± 0.6	0.963
n-6 PUFA intake	g	11.4 ± 2.7	11.6 ± 2.7	0.597
n-3 PUFA/n-6 PUFA		18.4 ± 3.2 (%)	18.1 ± 3.5 (%)	0.460
**Marital status**																				
Married		98.9	98.7	0.496
**Psychological distress at Mid-pregnancy^a^**																				
Yes		82.4	51.4	<0.001
**Alcohol intake**																				
>once/month		6.6	2.9	0.099
**Annual income (yen)**																				
Less than 4 million		11.0	6.3	0.073
4–8 million		39.6	31.7	
Over 8 million		39.6	53.2	
Missing		9.9	8.9	
**Maternal education**																				
Training school, high school, or less		19.8	17.3	0.606
Graduated college		20.9	18.3	
Graduated university		53.9	60.7	
Missing		5.5	3.7	
**Experience of giving birth**																				
Yes		39.6	34.7	0.368
**Normal delivery**																				
Yes		46.2	49.1	0.802
**Multiple fetuses**																				
Yes		4.4	1.9	0.137
**Gestation period**																				
Before 37 weeks		4.4	5.0	0.527

*^a^Diagnosed with Kessler-6 score (cutoff value of ≥9)*.

Crude and multivariate odds ratios (17) [95% confidence interval (CI)] for postpartum depression at 1 month after delivery across the quintile for fish and PUFA consumption are shown in Table 3. Median fish intake in the highest quintile (62.5 g per day) was about six times that of the lowest quintile (11.2 g per day) and median intake of EPA or DHA in the highest quintile (265.6 and 421.7 mg, respectively) was about eight times that of the lowest quintile (31.9 and 54.7 mg, respectively). There were no statistically significant associations between the risk of postpartum depression and the quintile of either fish or n-3 PUFA and n-6 PUFA intake.

**Table 3:** Odds ratios [95% confidence interval (CI)] for depression (EPDS score ≥9) at 1 month after childbirth according to quintile of fish and PUFA intake in late pregnancy.

			1 (low)	2	3	4	5 (high)	*p* for trend
Fish	g	Median intake	11.2	22.4	31.9	41.6	62.5	
		Case	41	43	35	38	34	
		Control	153	150	159	155	159	
		Crude model	1	1.07 (0.66–1.73)	0.82 (0.50–1.36)	0.91 (0.56–1.50)	0.80 (0.48–1.32)	0.288
		Multivariate model^a^	1	1.19 (0.70–2.02)	0.99 (0.57–1.71)	1.06 (0.62–1.81)	0.76 (0.44–1.31)	0.282

EPA	mg	Median intake	31.9	77.2	120.2	171.0	265.6	
		Case	44	41	33	35	38	
		Control	150	152	161	158	155	
		Crude model	1	0.92 (0.57–1.49)	0.70 (0.42–1.16)	0.76 (0.46–1.24)	0.84 (0.51–1.36)	0.315
		Multivariate model^a^	1	1.11 (0.66–1.88)	0.96 (0.56–1.67)	0.85 (0.50–1.47)	0.87 (0.51–1.47)	0.377

DHA	mg	Median intake	54.7	125.3	194.2	277.3	421.7	
		Case	46	40	32	35	38	
		Control	148	153	162	158	155	
		Crude model	1	0.84 (0.52–1.36)	0.64 (0.38–1.05)	0.71 (0.44–1.17)	0.79 (0.49–1.28)	0.240
		Multivariate model^a^	1	0.94 (0.56–1.59)	0.81 (0.47–1.40)	0.72 (0.42–1.24)	0.80 (0.47–1.35)	0.241

n-3 PUFA	g	Median intake	2.4	2.8	3.0	3.3	3.8	
		Case	37	38	47	32	37	
		Control	157	155	147	161	156	
		Crude model	1	1.04 (0.63–1.72)	1.36 (0.83–2.21)	0.84 (0.50–1.42)	1.01 (0.61–1.67)	0.749
		Multivariate model^a^	1	1.09 (0.63–1.88)	1.23 (0.72–2.11)	0.84 (0.48–1.49)	0.92 (0.53–1.60)	0.505

n-6 PUFA	g	Median intake	9.1	11.0	12.1	13.4	15.6	
		Case	34	37	47	44	29	
		Control	160	156	147	149	164	
		Crude model	1	1.12 (0.67–1.87)	1.50 (0.92–2.47)	1.39 (0.84–2.29)	0.83 (0.48–1.43)	0.882
		Multivariate model^a^	1	1.12 (0.64–1.96)	1.54 (0.90–2.64)	1.42 (0.83–2.45)	0.78 (0.44–1.41)	0.740

*EPDS, Edinburgh Postnatal Depression Scale; EPA, eicosapentaenoic acid; DHA, docosahexaenoic acid; PUFA, polyunsaturated fatty acid*.

*^a^Logistic regression adjusted for normal delivery, gestation period, multiple fetuses, parity, education, social class, age, marital status, pre-pregnancy BMI, psychological distress at mid-pregnancy, and energy intake at late pregnancy*.

Crude and multivariate ORs (95% CI) for postpartum depression at 6 months after delivery across the quintile for fish and PUFA consumption are shown in Table 4. Median fish intake and PUFA intake at 6 months after delivery did not differ to those at 1 month after delivery. Similar to the results at 1 month after delivery, there were no significant relationships between fish and PUFA intake and postpartum depression at 6 months after delivery.

**Table 4:** Odds ratios [95% confidence interval (CI)] for depression (EPDS score ≥9) at 6 months after childbirth according to quintile of fish and PUFA intake in late pregnancy.

Fish and fatty acids^a^			1 (low)	2	3	4	5 (high)	*p* for trend
Fish	g	Median intake	11.2	22.6	31.3	41.5	61.6	
		Case	21	17	15	20	18	
		Control	121	125	127	122	124	
		Crude model	1	0.78 (0.42–1.65)	0.53 (0.25–1.13)	1.00 (0.52–1.93)	0.89 (0.46–1.74)	0.812
		Multivariate model^b^	1	0.64 (0.31–1.33)	0.75 (0.35–1.60)	0.90 (0.44–1.84)	0.74 (0.36–1.53)	0.751

EPA	mg	Median intake	31.9	76.9	121.1	173.4	264.2	
		Case	21	16	14	24	16	
		Control	121	126	128	118	126	
		Crude model	1	0.73 (0.36–1.47)	0.63 (0.31–1.30)	1.17 (0.62–2.22)	0.73 (0.36–1.47)	0.874
		Multivariate model^b^	1	0.69 (0.33–1.46)	0.68 (0.31–1.47)	1.22 (0.61–2.43)	0.71 (0.34–1.47)	0.881

DHA	mg	Median intake	54.7	122.9	195.0	280.7	416.9	
		Case	21	17	13	22	18	
		Control	121	125	129	120	124	
		Crude model	1	0.78 (0.39–1.56)	0.58 (0.28–1.21)	1.06 (0.55–2.02)	0.84 (0.42–1.65)	0.937
		Multivariate model^b^	1	0.73 (0.35–1.53)	0.61 (0.28–1.33)	1.14 (0.57–2.30)	0.80 (0.39–1.65)	0.983

n-3 PUFA	g	Median intake	2.4	2.8	3.0	3.3	3.7	
		Case	19	20	21	11	20	
		Control	123	122	121	131	122	
		Crude model	1	1.06 (0.54–2.09)	1.12 (0.58–2.19)	0.54 (0.25–1.19)	1.06 (0.54–2.09)	0.579
		Multivariate model	1	1.20 (0.58–2.48)	1.14 (0.55–2.36)	0.50 (0.22–1.16)	1.13 (0.55–2.32)	0.544

n-6 PUFA	g	Median intake	9.1	10.9	12.1	13.4	15.3	
		Case	21	18	21	16	15	
		Control	121	124	121	126	127	
		Crude model	1	0.84 (0.42–1.65)	1.00 (0.52–1.93)	0.73 (0.36–1.47)	0.68 (0.34–1.38)	0.267
		Multivariate model^b^	1	0.88 (0.43–1.81)	1.01 (0.50–2.03)	0.75 (0.35–1.58)	0.65 (0.31–1.38)	0.242

*EPDS, Edinburgh Postnatal Depression Scale; EPA, eicosapentaenoic acid; DHA, docosahexaenoic acid; PUFA, polyunsaturated fatty acid*.

*^a^Logistic regression adjusted for normal delivery, gestation period, multiple fetuses, parity, education, social class, age, marital status, pre-pregnancy BMI, psychological distress at mid-pregnancy, energy intake at late pregnancy*.

## Discussion

Our study found that EPA and DHA intakes during late pregnancy did not significantly reduce the risk of postpartum depression both at 1 month after delivery and at 6 months after delivery among postpartum Japanese women, whose average dietary fish intake was higher than those of women in other countries (33, 34). As far as we know, this is the first prospective cohort study that evaluated the relationship between dietary intake of n-3PUFA at late pregnancy and measures of depressive symptoms with a focus on the time-points of 1 and 6 months after delivery.

Although several studies have attempted to clarify the relationship between n-3PUFA and prenatal depression (14, 35), only two prospective cohort studies have previously reported on dietary n-3PUFA consumption and postpartum depression (16, 17). Our findings were consistent with these two studies in that there was no association; however, in our study, we were able to be more specific regarding the time interval between effect of interest (fish and PUFA intake) and measurement of outcome (postpartum depression).

In these previous studies, the Japanese study collected information on dietary fish and n-3 PUFA intake throughout pregnancy, but not in specific gestational weeks (16), while in the other study a dietary survey was mailed to participants before 25 weeks’ gestation, but it did not include intake for specific gestational weeks (17). Middle or late gestation in pregnancy is an optimal time period to measure n-3 PUFA consumption, as intake is not affected with emesis at this time. Framing a shorter time interval for administration of the dietary survey helps to better clarify the relationship between n-3 PUFA intake and postpartum depression. Thus, in the present study, we limited the administration of sFFQ to gestational weeks’ 26–40.

Furthermore, the time-points used to conduct the EPDS survey in these two previous studies were markedly wide: from 2 to 9 months postpartum in Miyake’s study (16) and up to 1 year postpartum in Strom’s study (17). Postpartum depression is defined by the Diagnostic and Statistical Manual of Mental Disorders, Fifth Edition as depressive symptoms experienced 1 month after delivery (36). In Japan, the EPDS for postpartum depression was validated 1 month after delivery (37). On the other hand, it is reported that postpartum depression can develop as late as 3–7 months postpartum (38). In the present study, we were able to show a difference between the percentage of participants with a depression score at 1 month (19.8%) and at 6 months (12.8%) after delivery, and examine the association between n-3 PUFA and depression at both of these time-points.

In addition to the observational studies, several double-blind randomized controlled trials (RCTs) of n-3PUFA supplementation for postpartum depression have shown inconsistent results. Although one study reported that DHA supplementation of 300 mg from 24 to 40 weeks’ gestation prevented postpartum depression, study participants were limited to only 42 women (23). Two other RCTs with high-dose supplementation of EPA for 8 weeks suggested beneficial effects (39, 40). On the other hand, DHA supplementation of 220 mg/day from 16 weeks of pregnancy until 3 months postpartum did not prevent postpartum depression among 119 women at 6 months after delivery (20). As well, EPA+DHA supplementation of 800 mg/day at 26–36 weeks’ gestation showed no beneficial effect for postpartum depression among 2,339 women at 6 weeks and 6 months after delivery (21). Another recent RCT showed that EPA+DHA supplementation of more than 1,000 mg/day did not prevent postpartum depression from 6 to 8 weeks after delivery for women with a high risk of depression (≥9 of EPDS score in early pregnancy) (22).

Docosahexaenoic acid deficiency has been reported to be associated with serotonin, norepinephrine, and dopamine transmission dysfunction as well as those of neuronal membrane stability, leading to both mood disorders as well as cognitive dysfunction of depression (41). Furthermore, EPA reduces membrane arachidonic acid (an n-6PUFA) and prostaglandin E2 synthesis, both which are vital for the immune system to function and to maintain physical health, leading to somatic manifestations and physical comorbidity in depression when deficient (3). The role of n-3 PUFAs in immunity and mood function supports the promising hypothesis of the psychoneuroimmunology of depression (40). However, recent reports from epidemiological studies on the relationship between n-3PUFA levels of biological information and postpartum depression were inconsistent (18, 19, 42, 43). A study from Norway found that low EPA+DHA concentration in red blood cells in late pregnancy was associated with higher depression at 3 months after delivery (43) and a study from Australia reported that EPA and total n-3PUFA concentration of erythrocytes in late pregnancy were associated with postpartum depression (42). However, these studies did not conduct multivariate analysis for risk assessment.

Our sFFQ included 20 kinds of fish items and successfully captured a large variation of EPA and DHA intakes, despite being a cohort study with a reasonably large sample size. As there was a low percentage of participants taking EPA or DHA supplements in this study, our sample size did not decrease notably after exclusion of EPA or DHA supplement users. On the other hand, a high percentage of participants in this study had high levels of maternal education, high incomes, and were more health conscious with a low rate of smoking. Therefore, the response rate was relatively high and the sample size was sufficiently large, even after excluding current smokers and those respondents who gave incomplete or unclear answers. On the other hand, the dietary habits of the participants may not be representative of the average pregnant Japanese woman. Therefore, n-3PUFA may not deficient even in the group with the lowest intake. However, the median fish consumption of the lowest quintile was 11.2 g and that of the highest quintile was 62.5 g, compared to 23.1 g for the lowest quartile and 72.9 g for the highest quartile in Miyake’s study (16). It is possible that a ceiling effect may be in play when fish consumption is high among participants; however, as we included participants with a lower fish intake, our analysis provided more robust results. In addition, it is also possible that low-dose omega-3 PUFA was not effective for the prevention of postpartum depression.

This study also had several additional limitations. First, the EPDS that we used for screening postpartum depression is a widely used screening tool for postpartum depression around the world. Although the cut-off point may differ among countries or races, the reliability and validity of the cut-off point of nine for Japanese populations has been confirmed and the prevalence of postpartum depression was found to be comparable to Western countries (29). Study bias still remains because women with depression may not wish to complete the questionnaire. Second, at the beginning of the follow-up, 1,452 women answered a sFFQ, but the number of subjects included in the analysis was less than 1,000. In particular, there were 320 women who did not complete the EPDS. However, there was no difference in fish intake of at least subjects who completed EPDS and those who did not complete. Third, EPA and DHA intakes may relate to other healthy dietary habits, lifestyle, or residual factors. These potential biases might have confounded the results. Although we excluded participants who were diagnosed with psychiatric disorders before pregnancy, the diagnoses for these participants were made by medical doctors. However, we took into account that depressed mood during pregnancy was diagnosed with a Kessler-6 score (cutoff value of ≥9). Fourth, sFFQ is known to commonly over- or underestimate dietary intake, and these are most likely to bias true associations toward the null. Nevertheless, sFFQ used in this study was validated for the assessment of fish and n-3 PUFA comparing with both the dietary records and serum phospholipid. Fifth, in Japan, there is no recommended dietary allowance for n-3 and n-6 PUFA intake, and a Japanese sample might be biased due to high intake of fish oil, although not as high as those who are taking EPA or DHA supplement. Therefore, n-3 PUFA, as both dietary intake and as a supplement, might have protective effect for postpartum depression even among Japanese. Finally, fish and n-3PUFA intakes were only measured at one point during pregnancy, and the measurement time varied, taking place between gestational weeks 26 and 40, which limited our ability to clarify the effective period of n-3PUFA supplementation.

This prospective study found EPA and DHA intakes were not associated with the risk of postpartum depression at 1 month after delivery. Our findings suggest that dietary EPA and DHA without use of supplementation might not have a protective effect on postpartum depression. Future studies are required to further evaluate the relationship between n-3PUFA consumption and postpartum depression across other characteristics or other gestational periods of n-3 PUFA consumption.

## Ethics Statement

Ethics Committee at the National Center for Child Health and Development on August 2, 2010 (project number 417).

## Author Contributions

MK conceived of the research idea. RH, TF, KO, NM, and YT conducted the research. MK analyzed the data, wrote the paper, and had primary responsibility for the final content. All authors read and approved the final manuscript.

## Conflict of Interest Statement

The authors declare that the research was conducted in the absence of any commercial or financial relationships that could be construed as a potential conflict of interest.
